# Bias and Its Control in Stochastic Approaches to Electronic-Structure Theory

**DOI:** 10.1021/acs.jctc.5c01970

**Published:** 2026-03-17

**Authors:** Pavel Savchenko, Sayak Adhikari, Efrat Hadad, Eran Rabani, Roi Baer

**Affiliations:** † Fritz Haber Research Center for Molecular Dynamics, Institute of Chemistry, 98519 The Hebrew University of Jerusalem, Jerusalem 9190401, Israel; ■ Institute of Applied Physics, 98519 The Hebrew University of Jerusalem, Jerusalem 9190401, Israel; ‡ Department of Chemistry, University of California, Berkeley, and Materials Sciences Division, Lawrence Berkeley National Laboratory, Berkeley, California 94720, United States

## Abstract

Stochastic formulations of electronic-structure theory often reduce computational cost by replacing exact contractions with statistical estimates obtained from random samples, a procedure that inherently introduces random fluctuations and systematic bias. The fluctuations decay as *M* ^–1/2^ with the number of samples *M*, whereas the bias generated in nonlinear or self-consistent settings decays as *M* ^–1^ and can remain significant for moderate *M*. To control this bias we employ the jackknife-2 estimator, which reduces its leading term to $O\left(M^{-2}\right)$ with only modest extra cost. We examine bias formation and removal in three settings: (i) stochastic treatments of the Markovian master equation using bundled dissipators, (ii) stochastic Kohn–Sham density functional theory for warm dense hydrogen, and (iii) stochastic evaluation of the Hubbard-model partition function. The first two settings have been presented in earlier works; accordingly, we review them only briefly and focus primarily on the issue of bias control. The Hubbard-model application is entirely new. For this case, we present two approaches: a direct estimator, which has large variance but no bias, and a “midway transition probability” (ΣMTP) estimator, which has smaller variance but introduces bias. Applying the jackknife-2 procedure to the ΣMTP estimator controls this bias and yields a substantially lower total error than the direct estimator. Across all cases, jackknife bias removal markedly improves the accuracy and reliability of stochastic electronic-structure calculations without increasing the computational cost.

## Graphical abstract

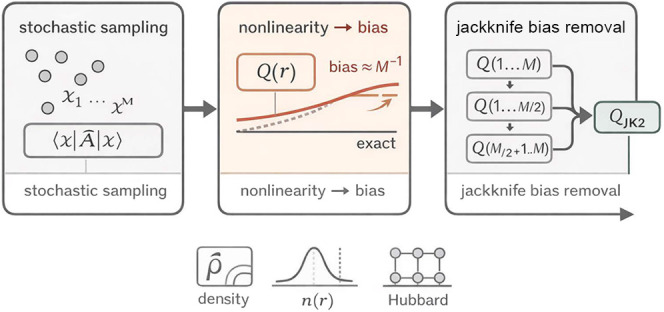

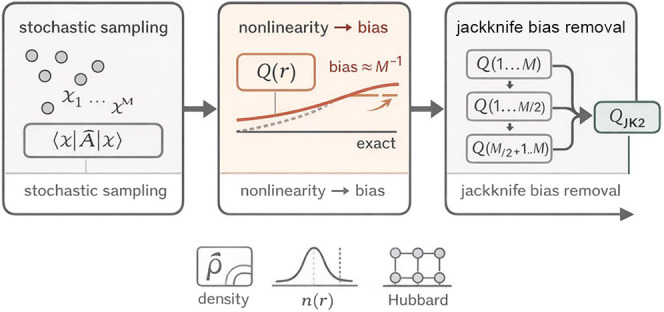

## 1 Introduction

Quantum chemistry applies quantum mechanics to describe matter at atomic and molecular scales, modeling electrons and nuclei as interacting quantum particles. Methods such as Hartree–Fock, perturbation and coupled cluster theories, Green’s-function approaches, and density functional theory (DFT) provide a hierarchy of approximations that connect microscopic dynamics to observable chemical properties. These deterministic frameworks have become essential for interpreting and predicting chemical phenomena, but their computational demand—often scaling steeply with system size—limits their applicability to large or strongly correlated systems.

Stochastic approaches to electronic-structure theory were developed in part by drawing on ideas that originated in the quantum Monte Carlo (QMC) framework.^1–8^ QMC demonstrated that random sampling can recover near-exact ground-state energies and correlation effects, and that auxiliary-field and full-configuration-interaction variants can systematically approach the exact solution.^9–12^ Motivated by these successes, related stochastic methodologies—most notably stochastic density-functional and Green’s-function approaches^13–22^ —have extended the paradigm beyond ground-state energetics to time-dependent and excited-state phenomena. These developments provide a unified and systematically improvable framework for large-scale electronic-structure calculations.

In all such stochastic approaches, the desired observable *q* is estimated statistically from *M* random samples, yielding an estimator *q̊*_*M*_ that converges to the exact value as *M* → ∞. Two error sources arise: random fluctuations, decaying as *M* ^–1/2^, and systematic bias, which typically scales as *M* ^–1^ (in Section 2 we provide a more detailed background for these concepts). While the fluctuations resemble familiar experimental uncertainties, the bias represents a reproducible offset that may persist and accumulate, particularly in long simulations or self-consistent cycles. For example, in stochastic DFT (sDFT), fluctuations in the atomic forces emulate the thermal noise required for Langevin dynamics and can therefore be used constructively,^8,23^ whereas any bias in these force estimators leads to systematic errors in resulting observables (see the last paragraph of Section 4 for a more detailed explanation). Understanding and mitigating systematic bias is therefore essential for ensuring the reliability of stochastic electronic-structure theory.

In this work, we analyze bias formation and correction in three representative contexts: (i) open quantum dynamics described by the Lindblad master equation,^24^ (ii) stochastic Kohn–Sham density-functional theory for warm dense matter,^13,18,25,26^ and (iii) two new stochastic evaluations of the Hubbard-model partition function, one of which is nonlinear. For each case, we formulate direct and jackknife estimators, quantify their accuracy and scaling behavior, and demonstrate practical bias suppression with minimal computational overhead.

The paper is organized as follows. In Section 2, we define the statistical concepts of bias and fluctuations and introduce the jackknife-2 estimator. Section 3 analyzes the bias in stochastic simulations of the Lindblad master equation. Section 4 examines bias propagation in stochastic Kohn–Sham density functional theory for warm dense matter. Section 5 presents a new stochastic method for the Hubbard model partition function, comparing the accuracy of direct and midway-transition-probability estimators. Finally, Section 6 provides a summary and concluding remarks.

## 2 Estimators, Fluctuations, Bias and the Jackknife-2 Technique

Here, we provide background material that serve as a basis for the unified view of the very different types of stochastic methods analyzed in this paper.

### 2.1 Statistical Errors in Estimation: Fluctuations and Bias

One introduces a random variable *r* and a smooth function *Q* such that applying *Q* to *M* independent and identically distributed (i.i.d.) samples *r*_1_,···,*r*_*M*_ from *r* produces a consistent estimator *q̊*_*M*_ = *Q*(*r*_1_,···, *r*_*M*_) for *q*. A consistent estimator is one for which the mean squared error $\mathrm{MSE}\left[{\mathrm{q̊}}_{M}\right]=E\left[{\left({\mathrm{q̊}}_{M}-q\right)}^{2}\right]$ vanishes in the limit *M* → ∞.

The MSE decomposes into two nonnegative terms, $\mathrm{MSE}\left[{\mathrm{q̊}}_{M}\right]={\mathrm{bias}}^{2}\left[{\mathrm{q̊}}_{M}\right]+V\left[{\mathrm{q̊}}_{M}\right]$ , where the bias, $\mathrm{bias}\left[{\mathrm{q̊}}_{M}\right]=E\left[{\mathrm{q̊}}_{M}\right]-q$ , measures systematic error, and the variance, $V\left[{\mathrm{q̊}}_{M}\right]=E\left[{\left({\mathrm{q̊}}_{M}-E\left[{\mathrm{q̊}}_{M}\right]\right)}^{2}\right]$ , quantifies statistical fluctuations. For a consistent estimator, both the bias and variance vanish separately as *M* → ∞.

In many cases, the target quantity *q* depends on the expected value μ=E[r] via a smooth function *Q*, so that *q* = *Q*(μ). The direct estimator is then defined as

$${\mathrm{q̊}}_{1>M}=Q\left({\mathrm{r̅}}_{1>M}\right),,{\mathrm{r̅}}_{j>k}=\frac{1}{k-j+1}\sum_{m=j}^{k}r_{m},k\geq j$$

By the central limit theorem, *r̅*_1>*M*_ approaches a normal random variable with mean $E{\mathrm{r̅}}_{1>M}=\mu$ and variance $V{\mathrm{r̅}}_{1>M}=E{\left({\mathrm{r̅}}_{1>M}-\mu \right)}^{2}=\frac{\sigma^{2}}{M}$ where $\sigma^{2}=V\left[r\right]$ . Expanding *Q* around μ using Taylor’s theorem, $Q\left({\mathrm{r̅}}_{1>M}\right)\approx Q\left(\mu \right)+Q^{\prime }\left(\mu \right)\left({\mathrm{r̅}}_{1>M}-\mu \right)+\frac{1}{2}Q^{\prime \prime }\left(\mu \right){\left({\mathrm{r̅}}_{1>M}-\mu \right)}^{2}+...$ , one finds, asymptotically,

1 $$\mathrm{bias}\left[{\mathrm{q̊}}_{1>M}\right]=\frac{1}{2}Q^{\prime \prime }\left(\mu \right)\frac{\sigma^{2}}{M}+O\left(M^{-3/2}\right)$$

and

2 $$V\left[{\mathrm{q̊}}_{1>M}\right]=\frac{{\left(Q^{\prime }\left(\mu \right)\sigma \right)}^{2}}{M}+O\left(M^{-3/2}\right)$$

From eq 1 we see that *q̊*_1*>M*_ is biased with the sign determined by *Q*″(μ) and the bias decreases as $O\left(M^{-1}\right)$ . From eq 2 the standard deviation of *q̊*_1>*M*_ decays as $O\left(M^{-1/2}\right)$ . For large *M*, the MSE is dominated by the variance; however, when *M* is moderate, the bias may play a significant role.

### 2.2 Jackknife-2 Estimator

The bias can be reduced by various methods. A simple and effective one is the jackknife-2 estimator,^27^ which generates three estimates: *q̊*_1>*M*_ obtained from the entire sample, ${\mathrm{q̊}}_{1>M/2}$ using half and ${\mathrm{q̊}}_{M/2+1>M}$ using the second half. The bias can then be estimated as $\frac{1}{2}\left[{\mathrm{q̊}}_{1>M/2}+{\mathrm{q̊}}_{M/2+1>M}\right]-{\mathrm{q̊}}_{1>M}$ and subtracting this from the best value yields the jackknife-2 estimator

3 $${\mathrm{q̊}}_{1>M}^{{\mathrm{jk}}_{2}}=2{\mathrm{q̊}}_{1>M}-\frac{1}{2}\left[{\mathrm{q̊}}_{1>M/2}+{\mathrm{q̊}}_{M/2+1>M}\right]$$

The bias of *q̊*_1>*M*_^*jk*_2_^ decreases asymptotically as $O\left(M^{-2}\right)$ . Thus, the jackknife estimator typically exhibits smaller bias and faster convergence than the direct estimator *q̊*_1>*M*_, though at roughly twice the computational cost if *Q* is expensive to evaluate. Its fluctuations are only mildly larger than those of the direct estimator.

## 3 Bias in the Stochastic Approach to the Markovian Master Equation

Real quantum systems are inherently open, continuously interacting with their surrounding environment. Consequently, their quantum-mechanical state cannot be determined with complete certainty. A common situation arises when we can specify a set of normalized kets, {|ϕ_α_⟩}, each representing a possible state of the system, occurring with a known probability π_α_ ≥ 0 (∑_α_π_α_ = 1). Such a situation is referred to as a *mixed state* and is formally represented by the density operator

$$\mathrm{\rho ̂}=\sum_{\alpha }\pi_{\alpha }\left|\phi_{\alpha }\right\rangle \left\langle \phi_{\alpha }\right|$$

The exact evolution of a mixed state is exceedingly complex: it requires solving the coupled dynamics of both the system and its macroscopic environment, and then projecting out the system’s reduced density matrix. A widely used approximation is the *Markovian Master Equation* (MME),^28–34^ developed independently by Lindblad^35^ and Gorini, Kossakowski, and Sudarshan.^36^ For a system with Hamiltonian $H_{\mathrm{sys}}$ , it takes the form

4 $$\frac{d}{\mathrm{dt}}\mathrm{\rho ̂}\left(t\right)=\frac{1}{i\hbar }\left[{Ĥ}_{\mathrm{sys}}+{Ĥ}_{\mathrm{Ls}},\mathrm{\rho ̂}\left(t\right)\right]+D\mathrm{\rho ̂}\left(t\right)$$

where $H_{\mathrm{Ls}}$ describes the Hermitean shift of the energy levels due to interaction with the environment, and the dissipator

5 $$D\mathrm{\rho ̂}\equiv \frac{1}{2}\sum_{b\in B}\gamma_{b}\left(\left[{\mathrm{L̂}}_{b}\mathrm{\rho ̂},{\mathrm{L̂}}_{b}^{†}\right]+\left[{\mathrm{L̂}}_{b},\mathrm{\rho ̂}{\mathrm{L̂}}_{b}^{†}\right]\right)$$

accounts for decoherence and relaxation of the system by the environment. Here, B indexes different dissipative channels, typically associated with the transition frequencies of ${Ĥ}_{\mathrm{sys}}$ . The functions γ_*b*_ are non-negative coupling rates, and the operators ${\mathrm{L̂}}_{b}$ are Lindblad or jump operators encoding system-environment interactions. The MME guarantees that the density operator remains positive and trace-preserving, as required for physical probability distributions. Although primarily justified for weak and Markovian system-environment coupling, the MME can also be extended to certain strong-coupling and/or non-Markovian scenarios by incorporating relevant environmental modes into the system Hamiltonian.^37–39^

Solving Markovian master equations (MMEs) for large quantum systems is computationally intensive, necessitating specialized software and algorithms.^40–43^ In the worst case, complexity scales with Hilbert space dimension *N* as $O\left(N^{5}\right)$ : $O\left(N^{2}\right)$ Lindblad operators and each operator multiplies the DM, a feat involving $O\left(N^{3}\right)$ operations. In many cases the Lindblad operators are sparse so the complexity drops to $O\left(N^{4}\right)$ .

To reduce computational scaling we use a vector $r={\left(r^{b}\right)}_{b\in B}$ with independent random variables, each having zero mean and unit variance

6 $$Er^{b}r^{b\prime *}=\delta_{\mathrm{bb}\prime }\to E{\mathrm{rr}}^{†}=I$$

Examples include *r* ^*b*^ ∈ {−1,1}, or *r* ^*b*^ = e^*iθ*_*b*_^ with θ_*b*_ randomly sampling the [0, 2π] interval. Using such a vector, we define the bundled Lindblad operator

$${\mathrm{R̂}}_{1}=\sum_{b\in B}r^{b}\sqrt{\gamma_{b}}{\mathrm{L̂}}_{b}$$

and the random bundled dissipator

7 $$D_{1}\mathrm{\rho ̂}\equiv \frac{1}{2}\left(\left[{\mathrm{R̂}}_{1}\mathrm{\rho ̂},{\mathrm{R̂}}_{1}^{†}\right]+\left[{\mathrm{R̂}}_{1},\mathrm{\rho ̂}{\mathrm{R̂}}_{1}^{†}\right]\right)$$

The full dissipator (([Disp-formula eq5])) is then expressed as a expected value over the bundled dissipator

8 $$D\equiv ED_{1}$$

To reduce statistical fluctuations, we can use a sample of *M* > 1 vectors **r**_*m*_, forming a sample of bundled Lindblad operators

9 $${\mathrm{R̂}}_{m}=\sum_{b\in B}\frac{r_{m}^{b}}{\sqrt{M}}\sqrt{\gamma_{b}}{\mathrm{L̂}}_{b}$$

with which the bundled dissipator is expressed as

10 $$D_{1>M}\mathrm{\rho ̂}=\frac{1}{2}\sum_{m=1}^{M}\left(\left[{\mathrm{R̂}}_{m}\mathrm{\rho ̂},{\mathrm{R̂}}_{m}^{†}\right]+\left[{\mathrm{R̂}}_{m},\mathrm{\rho ̂}{\mathrm{R̂}}_{m}^{†}\right]\right)$$

Here too $D=E\left[D_{1>M}\right]$ and fluctuations scale as *M* ^–1/2^.

The use of $D_{1>M}$ in the stochastic MME produces an estimator ρ̊_1···*M*_ by evolving from initial ρ̂(0)

11 $$\frac{d}{\mathrm{dt}}{\mathrm{\rho ̊}}_{1>M}\left(t\right)=\frac{1}{i\hbar }\left[H,{\mathrm{\rho ̊}}_{1>M}\left(t\right)\right]+D_{1>M}{\mathrm{\rho ̊}}_{1>M}\left(t\right)$$

Since the dissipator of eq 10 preserves the Lindblad form, this equation produces ρ̊_1>*M*_(*t*) which is completely positive and has its trace preserved (1) during the entire evolution.

However, the fluctuations in $D_{1.M}$ (∝*M* ^–1/2^) cause bias

12 $$\mathrm{bias}{\mathrm{\rho ̊}}_{1>M}\left(t\right)\equiv E{\mathrm{\rho ̊}}_{1>M}\left(t\right)-\mathrm{\rho ̂}\left(t\right)$$

due to nonlinear noise folding. Bias drops as *M* ^–1^ for large *M*.^44^ Jackknife estimators reduce bias faster (typically as *M* ^–2^)­

13 $${\mathrm{\rho ̊}}_{1>M}^{{\mathrm{jk}}_{2}}\left(t\right)=2{\mathrm{\rho ̊}}_{1>M}\left(t\right)-\frac{1}{2}\left({\mathrm{\rho ̊}}_{1>M/2}\left(t\right)+{\mathrm{\rho ̊}}_{M/2+1>M}\left(t\right)\right)$$

We demonstrate the method on a symmetric double-well oscillator of unit mass, whose Hamiltonian reads

14 $${Ĥ}_{\mathrm{sys}}=-\frac{1}{2}\frac{d^{2}}{dx^{2}}+{\left({\mathrm{x̂}}^{2}-\frac{9}{4}\right)}^{2}$$

We present the dynamics in the position representation using an equally spaced grid of *N*_*x*_ = 42 points spanning the interval [−2, 2] along the oscillator position *x* axis. The kinetic energy is represented on this grid via a standard 7-point finite-difference formula for the second derivative (see ref 24.). The potential *V*(*x*) = (*x* ^2^–9/4)^2^ consists of a squared parabola forming symmetric wells centered at *x* = ± 3/2 separated by a central barrier of height (9/4)^2^ at *x* = 0. A graphical depiction of the potential, with five lowest energy levels superimposed, is shown in Figure 1a. The ground and first excited states, with energies ε_1_ and ε_2_, lie well below the barrier maximum and exhibit a very small tunneling splitting due to the high barrier. The second excited state, with energy ε_3_, lies just below the barrier top.

**Figure 1:**
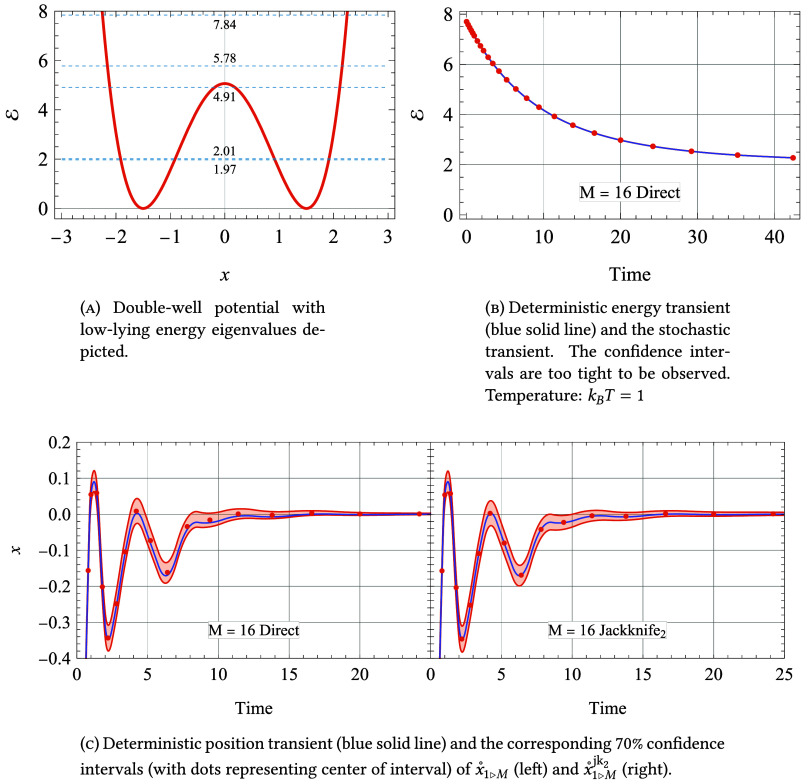
The double-well potential (panel A) and energy (panel B) and position (panel C) transients. All calculations use *M* = 16 bundled dissipators.

We use the Davies framework, briefly described in Appendix, for constructing the Lindblad operators coupling the oscillator to the environment at temperature *k*_B_ *T*, which, for this work is *k*_B_ *T* = 1. The coupling constants are taken from eq 30 with γ_*_ = 0.05 and ω_*c*_ = 1000. We start from a pure state of energy E=8 localized in the left well (negative values of *x*). The energy transient, shown in Figure 1b, exhibits a monotonic decay reaching a thermal value close to the ground state energy. During the energy relaxation, the oscillator oscillates and its position’s expected value, shown in Figure 1c even moves slightly to the right twice, as it settles into its thermal value of zero.

In Figure 1 we show the deterministic and stochastic (with an *M* = 16 bundled dissipator) energy and position transients. For the energy, the stochastic and deterministic results are essentially indistinguishable on the scale of the figure. For the position, a noticeable 70% confidence interval (CI) appears as a shaded region surrounding the deterministic curve. In the left panel, the CI is centered on *x̊*_1>*M*_, while in the right it is centered on *x̊*°_1>*M*_^jk_2_^. We verified that the CI width at each time point follows statistical scaling, being proportional to 1/√*M*.

The deviation of the estimate *x̊* (*t*) from the deterministic value *x*(*t*) defines the bias, which is visibly smaller for the jackknife estimates *x̊*_1 > *M*_^jk_2_^ than for *x̊*_1>*M*_. However, in both cases the bias remains well below the CI width and is therefore negligible. As shown in Section 2, the bias in *q̊*_1>*M*_ decreases asymptotically as $O\left(M^{-1}\right)$ , while for the jackknife estimate *q̊*_1>*M*_^jk_2_^ it decreases as $O\left(M^{-2}\right)$ . This behavior is confirmed for the energy in Figure 2; for the position, the trend is less clear – likely because *M* is not yet large enough. Estimating the bias accurately at such small values is challenging, as it would require prohibitively large sampling.

**Figure 2:**
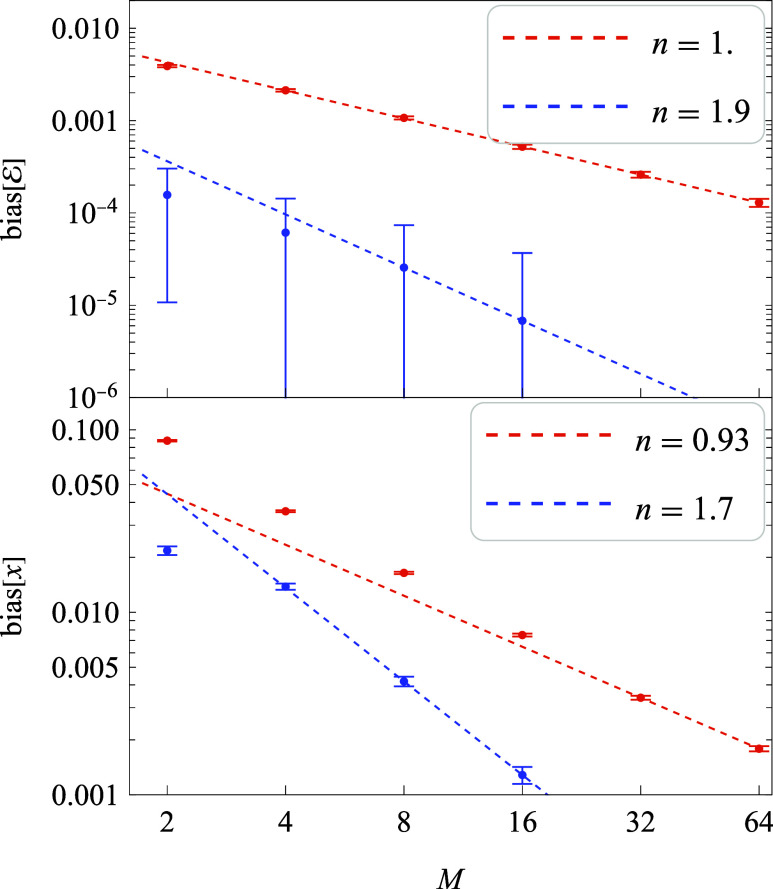
Bias in energy and position estimates at *t* = 9.5 vs *M*. Red (blue) symbols: direct (jackknife) estimates, averaged over 18,000 runs. Dashed lines show fits of the form *A*/*M* ^*n*^, using the three largest-*M* points in each series (the fitted *n* values appear in the legends).

## 4 Stochastic Density Functional Theory

The Kohn–Sham density functional theory (KS-DFT)^45,46^ is a framework for obtaining the electron density *n*(**r**) of a molecular system. It is based on solving a one-electron Hamiltonian, the Kohn–Sham Hamiltonian

15 $${ĥ}_{\mathrm{KS}}\left[n\right]=-\frac{1}{2}\nabla^{2}+{\mathrm{v̂}}_{\mathrm{nl}}+v_{\mathrm{loc}}\left(r\right)+v_{H}\left[n\right]\left(r\right)+v_{\mathrm{xc}}\left[n\right]\left(r\right)$$

where *v̂*_nl_ + *v*_loc_(**r**) are the nonlocal and local pseudopotentials describing electron nucleus interactions, $v_{H}\left[n\right]\left(r\right)=\int \frac{n\left(r^{\prime }\right)}{\left|r-r^{\prime }\right|}dr^{\prime }$ is the Hartree potential accounting for classical electron electron repulsion, and *v*_*xc*_[*n*]­(**r**) is the exchange correlation potential incorporating quantum exchange and correlation effects.

For a system with *N*_*e*_ electrons, the density is obtained as

16 $$n\left(r\right)=2\sum_{n=1}^{N_{e}/2}{\left|\psi_{n}\left(r\right)\right|}^{2}$$

where ψ_*n*_(**r**) are the lowest-energy eigenstates of *ĥ*_KS_. The KS procedure iterates toward a fixed-point density: the density *n*(**r**) used to build *ĥ*_KS_ is the same as that obtained from its eigenstates. The theory also provides total forces on nuclei, which can be used in classical molecular dynamics (MD) simulations. Commonly, Nos–Hoover or Langevin thermostats are added to sample the canonical ensemble at temperature *T*, allowing first-principles determination of material equations of state.^23,47^

KS-DFT calculations scale as $O\left(N_{e}^{3}\right)$ and are therefore computationally expensive for large systems containing thousands of atoms. Such systems are especially relevant near phase transitions or when mixtures are considered. A stochastic approach to DFT (sDFT) offers an alternative by estimating, rather than explicitly computing, the density and force predictions of KS-DFT using statistical sampling.

Stochastic density functional theory (sDFT)^13,17,18,25,48,49^ combines two notions:

1. **Finite-temperature Kohn**–**Sham DFT**: At inverse temperature β = (*k*_B_ *T*)^−1^ and chemical potential μ, the KS-DFT electron density can be expressed as
17 $$n\left(r\right)=2\left\langle r\left|{\mathrm{F̂}}_{\beta ,\mu }^{2}\right|r\right\rangle$$
  where $2{\mathrm{F̂}}_{\beta ,\mu }^{2}=2F_{\beta ,\mu }{\left({ĥ}_{\mathrm{KS}}\right)}^{2}$ is the finite temperature Kohn–Sham density operator with
$$F_{\beta ,\mu }{\left(\varepsilon \right)}^{2}=\frac{1}{1+e^{\beta \left(\varepsilon -\mu \right)}}$$
  the Fermi–Dirac function. Equation 17 can also be written as an operator trace
18 $$n\left(r\right)=2\mathrm{Tr}\left[{\mathrm{F̂}}_{\beta ,\mu }\left|r\right\rangle \left\langle r\right|{\mathrm{F̂}}_{\beta ,\mu }\right]$$
  Since ∫|r⟩⟨r|dr=I (the identity operator), the average number of electrons is $N_{e}=2\mathrm{Tr}\left[{\mathrm{F̂}}_{\beta ,\mu }^{2}\right]$ .
2. **Stochastic trace formula**: The stochastic algorithm comes in through the stochastic trace formula,^50^ expressing the trace of any operator *Â* an expectation value of a random variable
19 TrÂ=E[⟨χ|Â|χ⟩]
  where |χ⟩ is a random ket satisfying E[|χ⟩⟨χ|]=1 . In practice, the trace is estimated using a sample of *M* independent random kets |χ_*m*_⟩
20 $${Å}_{1>M}=\frac{1}{M}\sum_{m=1}^{M}\left\langle \chi_{m}\left|Â\right|\chi_{m}\right\rangle ,E\left[\left|\chi_{n}\right\rangle \langle \chi_{n\prime }|\right]=1\delta_{\mathrm{nn}\prime }$$

Combining eqs 17 and 19 gives the defining relations for sDFT

$$\left|\xi \left[n\right]\right\rangle ={\mathrm{F̂}}_{\beta \mu }\left[n\right]\left|\chi \right\rangle ,n\left(r\right)=E{\left|\left\langle r|\xi \left[n\right]\right\rangle \right|}^{2}$$

In practice, the expectation value is estimated by a finite sample average of *M* random kets |χ_*m*_⟩

$$\left|\xi_{m}\left[{\mathrm{n̊}}_{j>k}\right]\right\rangle ={\mathrm{F̂}}_{\beta ,\mu }\left[{\mathrm{n̊}}_{j>k}\right]\left|\chi_{m}\right\rangle ,,{\mathrm{n̊}}_{j>k}\left(r\right)=\frac{1}{k-j+1}\sum_{m=j}^{k}{\left|\left\langle r|\xi_{m}\left[{\mathrm{n̊}}_{j>k}\right]\right\rangle \right|}^{2}$$

The basic sDFT density estimator, *n̊*_1>*M*_(**r**), uses the full sample. Unfortunately, replacing the true expected value with a finite-sample average introduces bias

21 $$E{\mathrm{n̊}}_{1>M}\left(r\right)\neq n\left(r\right)$$

which propagates to all derived observables *q̊*_1>*M*_. The partial sample estimators ${\mathrm{n̊}}_{1>M/2}\left(r\right)$ and ${\mathrm{n̊}}_{M/2+1>M}\left(r\right)$ can be used to compute corresponding observables ${\mathrm{q̊}}_{1>M/2}$ and ${\mathrm{q̊}}_{M/2+1>M}$ and the three estimates can be combined according to eq 13 to reduce the bias.

We demonstrate this bias and the associated fluctuations for four warm dense hydrogen systems—H_8_ and H_128_ at densities of 1 and 2 g cm^–3^. We first describe the deterministic KS reference results, summarized in Table 1, which lists the corresponding energies and pressures. Because such calculations aim to approach the thermodynamic limit—i.e., properties independent of the simulation cell size—we also report results for H_256_ to assess finite-size effects. The eight-atom system is clearly too small: at 1 g cm^–3^ it exhibits nearly a 1.5-fold increase in pressure when enlarged to 128 atoms. In contrast, the 128 and 256 atom systems yield similar energies and pressures. At the higher density of 2 g cm^–3^, even the 128-atom system is not fully converged. Comparing the two densities, the energies decrease with increasing density (reflecting stronger electron electron repulsion), while the pressures rise sharply—by roughly a factor of 5.

**Table 1:** Deterministic Single-Point Kohn–Sham (KS) Energies (*E*_*KS*_) and Pressures (*P*) for the H_8_, H_128_ and H_256_ systems at *T* = 15,000 K and Densities of 1 and 2 g/cm^3^ ^a^

density	1 g/cm^3^			2 g/cm^3^		
system	H_8_	H_128_	H_256_	H_8_	H_128_	H_256_
*L*(Å)	2.37	5.98	7.52	1.88	4.75	5.98
*E*_*KS*_ (eV/e)	–14.6	–13.9	–13.6	–12.3	–10.9	–10.3
*P*(GPa)	159	242	242	990	1240	1310

a The DFT calculations used simulation cells of size *L* (shown) and employed the Baldereschi sampling point with a plane-wave cutoff energy of 816 eV. The nuclear coordinates of these systems are provided in the Supporting Material.

Table 2 summarizes the statistical errors of sDFT relative to deterministic KS-DFT for both densities and two sample sizes (*M* = 10 and 40). At 1 g cm^–3^, using *M* = 10 stochastic kets, the KS energy shows small relative biases of −1.2% for H_8_ and −2.0% for the 16-fold larger H_128_. Increasing to *M* = 40 reduces these biases by approximately a factor of 4, consistent with the expected 1/*M* scaling. The jackknife correction further suppresses the bias by an order of magnitude or more, to below 0.1% in all cases. At the higher density (2 g cm^–3^), the energy biases are somewhat larger, yet the jackknife remains equally effective.

**Table 2:** Statistical Errors of the sDFT Estimation (Relative to Deterministic KS-DFT) in the Systems Described in Table 1 ^a^

*q*	*M*	err. typ	1 g/cm^3^				2 g/cm^3^			
			H_8_		H_128_		H_8_		H_128_	
			*q̊*_1>*M*_	*q̊*_*M*_^jk^	*q̊*_1>*M*_	*q̊*_*M*_^jk^	*q̊*_1>*M*_	*q̊*_*M*_^jk^	*q̊*_1*>M*_	*q̊*_*M*_^jk^
*E*_*KS*_ (%)	10	bias	–1.2	–0.085	–2.0	–0.11	–2.5	–0.54	–3.3	–0.16
		STD	4.0	5.6	1.8	2.6	6.4	9.1	3.2	4.5
		RMSE	4.2	5.6	2.7	2.6	6.9	9.2	4.6	4.5
	40	bias	–0.33	–0.04	–0.49	0.0071	–0.79	–0.19	–0.84	–0.014
		STD	2.0	2.8	0.89	1.3	3.0	4.3	1.5	2.2
		RMSE	2.0	2.8	1.0	1.3	3.1	4.4	1.8	2.2
*P* (%)	10	bias	9.3	3.0	5.6	0.28	5.1	2.3	2.7	0.14
		STD	22	25	5.8	6.4	9.4	11	3.2	3.4
		RMSE	24	25	8.1	6.4	11	11	4.2	3.4
	40	bias	3.5	1.0	1.4	–0.0014	2.2	1.0	0.72	0.015
		STD	11	12	3.0	3.1	4.7	5.1	1.6	1.6
		RMSE	12	12	3.4	3.1	5.2	5.2	1.8	1.6
*F*eV/Å	10	MAB	0.050	0.038	0.039	0.005	0.10	0.095	0.046	0.0082
	40	MAB	0.023	0.011	0.0095	0.0020	0.061	0.044	0.014	0.0042

a Shown, are the relative (%) bias, standard deviation (STD) and root mean square error (RMSE) of the KS energy *E*_KS_ and pressure *P*, together with the mean absolute bias (MAB) of the force *F* (averaged over all atoms and Cartesian components). Results are presented for sDFT with 10 and 40 stochastic orbitals.

The stochastic fluctuations, reflected in the standard deviation (STD), are significantly larger than the mean biases. For the KS energy, the STD at 1 g cm^–3^ and *M* = 10 is about 4% for H_8_ and 2% for H_128_; increasing *M* to 40 reduces these fluctuations roughly by a factor of 2, consistent with the expected 1/√*M* behavior. Similar trends are seen at the higher density, although the relative fluctuations there are slightly larger. For the pressure, the stochastic variability is an order of magnitude larger than for the energy: at *M* = 10, the STD reaches 20–25% for H_8_ and 6% for H_128_, decreasing by about a factor of 2 at *M* = 40. The jackknife procedure does not substantially reduce the fluctuations—its effect is mainly on the bias—though it stabilizes the overall estimates.

The resulting root-mean-square errors (RMSE) combine both bias and fluctuation effects. For the KS energy, the RMSE values are close to the STD, confirming that the statistical uncertainty dominates over the systematic bias. At *M* = 10, the energy RMSE is 4% for H_8_ and 3% for H_128_; for *M* = 40, it drops to roughly 2 and 1%, respectively. For the pressure, the RMSE remains much larger— 20–25% for H_8_ and 8% for H_128_ at *M* = 10—and improves to about half those values at *M* = 40. At 2 g cm^–3^, both pressure fluctuations and RMSE values are noticeably smaller, suggesting that higher density leads to smoother statistical sampling.

The mean absolute bias (MAB) of the forces follows a similar pattern. For H_8_, the MAB decreases from 0.050 to 0.023 eV/Å as *M* increases from 10 to 40, while for H_128_ it drops from 0.039 eV/Å to below 0.010 eV/Å. The jackknife consistently improves the force estimates, reducing the MAB by factors of three to five for the small system and by an order of magnitude for the larger one.

The analysis concludes that in most calculations, bias is secondary to fluctuations, which dominate the RMSE. Since bias decreases as *M* ^–1^ while fluctuations scale as *M* ^–1/2^, increasing *M* further tilts the error budget toward fluctuations. This holds for a single sDFT run. However, sDFT is typically employed within molecular dynamics simulations to sample the canonical distribution via a Langevin equation. In this context, fluctuations are balanced through fluctuation–dissipation relations^8,23^ with a properly selected friction coefficient^18^ and are therefore not intrusive. Because molecular dynamics involves many time steps, fluctuations average out while any persistent bias accumulates. For pressure, the bias already contributes significantly to the total error, and its relative role is amplified in MD. Therefore, bias removal in sDFT, especially with a limited number of stochastic orbitals, is essential for obtaining accurate ensemble averages.

## 5 Quantum Monte Carlo for the Partition Function in the Hubbard Model

The Hubbard model Hamiltonian, for *L* single particle states is

22 $$Ĥ=\mathrm{T̂}+Û=-t\sum_{\sigma =↑↓}\sum_{\left\langle \mathrm{ij}\right\rangle }{â}_{i\sigma }^{†}{â}_{j\sigma }+U\sum_{i}{\mathrm{n̂}}_{i↑}{\mathrm{n̂}}_{i↓}$$

where *n̂*_*iσ*_: = *â*_*i*σ_^†^ *â*_*i*σ_, *i* = 1,···,*L*, and ⟨*ij*⟩ are nearest neighbor sites (*i* ≠ *j*) and *t* and *U* are non-negative parameters. The goal is to calculate the partition function for *N*_↑_ and *N*_↓_ electrons

23 $$Z=\mathrm{Tr}\left[e^{-Ĥ\beta }\right]$$

where the trace is over all *single-configuration kets*

$$\left|n\right\rangle =\left|n_{1↑}\cdot \cdot \cdot n_{L↑},n_{1↓}\cdot \cdot \cdot n_{L↓}\right\rangle ≔{\left({â}_{1↑}^{†}\right)}^{n_{1↑}}\cdot \cdot \cdot {\left({â}_{L↑}^{†}\right)}^{n_{L↑}}{\left({â}_{1↓}^{†}\right)}^{n_{1↓}}\cdot \cdot \cdot {\left({â}_{1↓}^{†}\right)}^{n_{L↓}}\left|0\right\rangle$$

where |0⟩ is the vacuum state (no electrons) and *n*_*i*σ_ (σ = ↑or ↓) is the occupation of the spin-site, either 0 or 1. We also note ∑_*i*σ_ *n*_*i*σ_ = *N*_*e*_ is the total number of electrons.

Using the single-configuration kets as basis the partition function can be written as

24 $$Z=\sum_{n}\left\langle n\left|\underset{K}{\underset{︸}{e^{-Ĥ\Delta \beta }\;\cdot \cdot \cdot e^{-Ĥ\Delta \beta }\;}}\right|n\right\rangle$$

where the summation is over all the relevant single configuration kets. The exponential $e^{-Ĥ\beta }$ is expressed as a product of *K* steps $\underset{K}{\underset{︸}{e^{-Ĥ\Delta \beta }\;\cdot \cdot \cdot e^{-Ĥ\Delta \beta }\;}}$ , $\left(\Delta \beta =\frac{\beta }{K}\right)$ .

### 5.1 The Partition Function as a Random Walk

For each single-configuration ket |**n**⟩, we set |**n**_0_⟩≔|**n**⟩ and using the stochastic process described in Appendix we sample a random single-configuration ket |**n**_1_⟩*a*_**n**,**n**_1__ where

25 $$E\left(\left|n_{1}\right\rangle a_{n,n_{1}}\right)=e^{-Ĥ\Delta \beta }\left|n_{0}\right\rangle$$

The method to randomly sample |**n**_1_⟩*a*_**n**,**n**_1__ is described in Appendix. Note the crucial fact that both |**n**_0_⟩ and |**n**_1_⟩ are single-configuration kets. The partition function is now

$$Z=E\sum_{n}\left\langle n\left|\underset{K-1}{\underset{︸}{e^{-Ĥ\Delta \beta }\;\cdot \cdot \cdot e^{-Ĥ\Delta \beta }\;}}\right|n_{1}\right\rangle a_{n,n_{1}}$$

where we pulled the expected value symbol out of the integrals and summations, since it is a linear operation.

Given the single configuration ket |**n**_1_⟩, we can use our stochastic propagation scheme to find a single-configuration ket |**n**_2_⟩ and the appropriate amplitude *a*_**n**_1_, **n**_2__ for which the expected value of |**n**_2_⟩*a*_**n**_1_, **n**_2__ is equal to $e^{-Ĥ\Delta \beta }\left|n_{1}\right\rangle$ . We thus have for the partition function

$$Z=E\sum_{n}\left\langle n\left|\underset{K-2}{\underset{︸}{e^{-Ĥ\Delta \beta }\;\cdot \cdot \cdot e^{-Ĥ\Delta \beta }\;}}\right|n_{2}\right\rangle a_{n,n_{1}}a_{n_{1},n_{2}}$$

where now E is taken as the combined expected value of the first and second stochastic propagation steps, described in Appendix.

We continue this scheme *K* times arriving at a final single-configuration ket |**n**_*K*_⟩, and therefore

$$Z=E\sum_{n}\left\langle n|n_{K}\right\rangle a_{n,n_{1}}\cdot \cdot \cdot a_{n_{K-1},n_{K}}$$

### 5.2 The Direct Estimator

When evaluating this expression using a finite sample of *I* = 1,···,*M* |**n**_*K*_⟩’s and transition amplitudes

$${\left\langle n|n_{K}\right\rangle }^{\left(M\right)}≔\frac{1}{M}\sum_{I=1}^{M}\left\langle n|n_{K}^{I}\right\rangle a_{n,n_{1}}^{I}\cdot \cdot \cdot a_{n_{K-1},n_{K}}^{I}$$

The “direct estimator” is

26 $${\mathrm{Z̊}}_{M}^{\mathrm{direct}}≔\sum_{n}{\left\langle n|n_{K}\right\rangle }^{\left(M\right)}$$

and clearly $Z=E{\mathrm{Z̊}}_{M}^{\mathrm{direct}}$ , which has no bias. However, since ⟨**n**|**n**_*K*_⟩^*I*^ is zero unless |**n**_*K*_⟩= |**n**⟩, this estimator has a large variance, especially when *K* and the dimension of the Hilbert space are large.

### 5.3 Midway Transition Probabilities

To reduce variance we insert a resolution of the identity midway in the propagation, expressing the partition function as a sum over the midway transition probabilities (MTPs)

27 $$Z=\sum_{n}\left\langle n\left|e^{-Ĥ\beta /2}e^{-Ĥ\beta /2}\right|n\right\rangle =\sum_{n,n^{\prime }\;}{\left|\left\langle n\left|e^{-Ĥ\beta /2}\right|n^{\prime }\right\rangle \right|}^{2}$$

We use the stochastic propagation to create random variables *A*_**n**,**n**′_ which have, as expectation values, the midway transition amplitudes

28 $$EA_{n,n\prime }=\left\langle n\left|e^{-Ĥ\beta /2}\right|n^{\prime }\right\rangle$$

Squaring these and summing over the MTPs, yields

29 $$Z=\sum_{n,n^{\prime }\;}{\left|EA_{n,n\prime }\right|}^{2}$$

When evaluating this expression using a finite sample

$${\mathrm{A̅}}_{n,n\prime }^{\left(M\right)}≔\frac{1}{M}\sum_{I=1}^{M}A_{n,n\prime }^{I}$$

each of the *A*_**n**,**n**′_^*I*^ has the property of eq 28, from which

$$E{\mathrm{A̅}}_{n,n\prime }^{\left(M\right)}=\left\langle n\left|e^{-Ĥ\beta /2}\right|n^{\prime }\right\rangle ,,V{\mathrm{A̅}}_{n,n}^{\left(M\right)}=\frac{\sigma_{n,n}^{2}}{M}$$

where $\sigma_{n,n\prime }^{2}=VA_{n,n\prime }^{I}$ .

### 5.4 The MTP Estimator

Since $Z=\sum_{n,n^{\prime }\;}{\left|E{\mathrm{A̅}}_{n,n\prime }^{\left(M\right)}\right|}^{2}$ (an equivalent expression to eq 29) holds, we take the sum over the average MTPs

$${\mathrm{Z̊}}_{M}^{\Sigma \mathrm{MTP}}≔\sum_{n,n^{\prime }\;}{\left|{\mathrm{A̅}}_{n,n\prime }^{\left(M\right)}\right|}^{2}$$

as estimator of Z . This estimator, however, is biased, since by standard statistics

$$E{\mathrm{Z̊}}_{M}^{\Sigma \mathrm{MTP}}=Z+\frac{1}{M}\sum_{n,n^{\prime }\;}\sigma_{n,n\prime }^{2}$$

and the second term on the right is the bias, diminishing as *M* ^–1^. Under these circumstances we expect the jackknife-2 estimator, eq 3, with $Z^{\Sigma \mathrm{MTP}}$ replacing *q*, to offer better control of the bias.

### 5.5 Role of Bias Control

We evaluate the statistical performance of the direct and ΣMTP methods by calculating the relative root-mean-square error (RMSE) and bias as a function of the number of samples *M*. For these tests, we utilize a Hubbard Hamiltonian [eq 22] with *L* = 6 sites and hopping parameter *t* = 1. To isolate the sampling behavior from discretization errors, reference values are obtained from numerically exact traces of the propagator with Δβ = 0.05 and *K* = 200.

As shown in Figure 3a,b, the direct approach is more efficient at low sampling because it is inherently unbiased. In contrast, the ΣMTP methods exhibit a large initial bias. However, because the direct method’s error is purely fluctuational, it decays slowly as *M* ^–1/2^. While the ΣMTP method benefits from smaller fluctuations—since it does not require returning to the initial state—its significant bias initially prevents it from outperforming the direct approach, even though this bias drops at a faster rate of *M* ^–1^ (Panels c,d).

**Figure 3:**
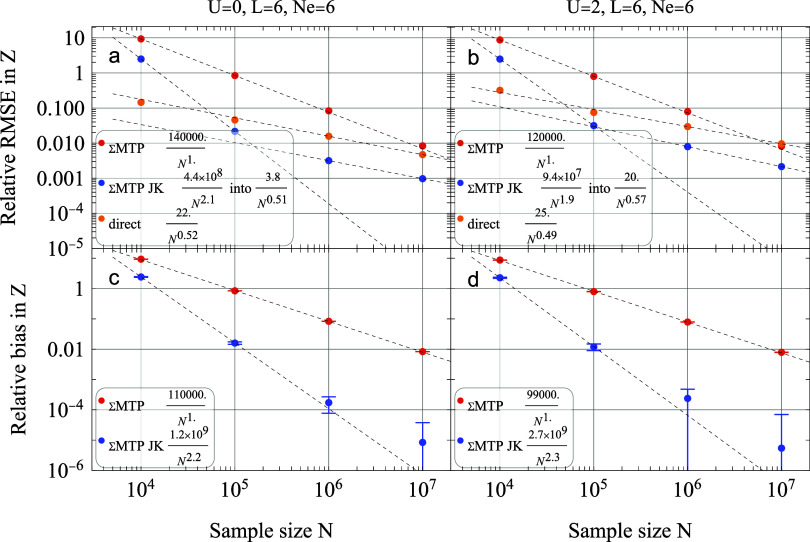
Relative root-mean-square error (panels a and b) and bias (panels c and d) as functions of the sample size *M* for partition-function estimates. The results compare the direct approach [eq 26], the ΣMTP approach [eq 29], and the ΣMTPJK (Jackknife) estimator for *U* = 0 and 2. Calculations were performed at half-filling (*N*_*e*_ = 6) and β = 1. Reference values are calculated using exact traces of the Trotterized operator to ensure that the errors shown represent sampling statistics rather than finite time-step effects.

The inclusion of the Jackknife estimator in ΣMTP-JK effectively resolves this limitation by removing the leading-order bias, which then scales as *M* ^–2^. Consequently, the ΣMTP-JK results achieve a total error nearly five times smaller than the direct method. Notably, these scaling relationships and the resulting efficiency gains appear robust against changes in the on-site interaction parameter *U*.

## 6 Discussion and Conclusions

Stochastic formulations of electronic-structure theory offer scalable alternatives to deterministic quantum-chemical methods, which often become prohibitively expensive for large, warm, or strongly correlated systems. Their replacement of exact contractions with random sampling introduces two types of statistical errors: fluctuations, which decay as *M* ^–1/2^, and systematic bias, which decays as *M* ^–1^ but may accumulate in nonlinear or self-consistent settings. Here *M* denotes the number of stochastic samples (e.g., bundled dissipators, stochastic orbitals, or stochastic trajectories). In this work we examined how these errors arise and propagate in three representative settings—stochastic open-system dynamics, stochastic density functional theory, and stochastic evaluation of the Hubbard-model partition function—and showed how estimator design and bias-removal strategies govern the accuracy and reliability of the resulting predictions.

For the stochastic Markovian master equation, bundled dissipators provide an unbiased representation of the Lindblad dissipator but introduce fluctuations whose nonlinear folding during time propagation produces a small residual bias in the density matrix. This bias remains well below the statistical confidence intervals for modest *M*∼10, and the jackknife-2 estimator further reduces it at roughly double the computational cost. It also significantly improves the scaling of bias from $O\left(M^{-1}\right)$ to $O\left(M^{-2}\right)$ .

In stochastic density functional theory (sDFT), bias plays a more significant role. Because the Kohn–Sham map is nonlinear and self-consistent, finite sampling induces systematic shifts in the electron density that propagate to energies, pressures, and forces. For warm dense hydrogen, fluctuations dominate the error of a single sDFT calculation, but in Langevin molecular-dynamics the forces are calculated over and over again, with fluctuations serving as physical thermal noise. Therefore, while fluctuations are not a problem, bias in the force estimation degrades predictions. The jackknife-2 estimator again proves effective: it reduces the bias in energies and pressures by more than an order of magnitude and is essential for obtaining reliable thermodynamic quantities at moderate *M*.

For the Hubbard-model partition function, the direct estimator is unbiased but has large fluctuations scaling as *M* ^–1/2^, whereas the ΣMTP estimator exhibits lower variance but a substantial positive bias scaling as *M* ^–1^. The jackknife-2 estimator removes the leading bias term and yields a ΣMTPJK estimator whose total error scales as *M* ^–1^–*M* ^–2^, outperforming the direct method by up to a factor of 5 in the examples studied, with little sensitivity to the interaction strength *U*.

The utility of bias removal extends to mixed deterministic/stochastic schemes, such as the partially stochastic resolution-of-the-identity approaches.^22^ Whenever stochastic estimates are fed into nonlinear functionals—for instance, in constructing self-energies or correlation functions—bias arises. The jackknife-2 correction, which relies only on the analytic structure of the bias scaling, applies directly in these contexts. It therefore offers a general, computationally inexpensive tool to enhance the precision of hybrid stochastic electronic structure methods.

Overall, bias is not a minor correction but depends strongly on context, especially when stochastic estimates feed into nonlinear procedures or long-time simulations. Simple, general techniques such as the jackknife-2 estimator efficiently suppress bias and are most valuable when estimator nonlinearities are intrinsic, as in sDFT or ΣMTP quadratures. With appropriate bias-removal strategies, stochastic methods achieve significantly higher accuracy without increasing the number of samples, reinforcing their value as reliable and efficient tools for large, warm, and strongly correlated quantum systems.

## Supplementary Material









## Davies Recipe

We employ the Davies approach to construct the Lindblad operators, where the environment (with Hamiltonian ${Ĥ}_{\mathrm{env}}$ ), in thermal equilibrium at temperature *T*, and the system are combined in a total Hamiltonian

$${Ĥ}_{\mathrm{tot}}={Ĥ}_{\mathrm{sys}}+{Ĥ}_{\mathrm{env}}+X⊗Y$$

where the last term represents the system-environment coupling, with X and Y denoting system and environment operators, respectively.

The Lindblad operators are then defined by Davies as

$${\mathrm{L̂}}_{\omega }=\sum_{n,m}\left|n\right\rangle \left\langle n\right|\mathrm{x̂}\left|m\right\rangle \left\langle m\right|\delta_{\omega -\left(\varepsilon_{m}-\varepsilon_{n}\right)}$$

where ω are the Bohr frequencies of the system, i.e., the energy differences ε_*n*_ – ε_*m*_ between the eigenvalues ε_*n*_ of ${Ĥ}_{\mathrm{sys}}$ . The states |*n*⟩ are the corresponding eigenstates of ${Ĥ}_{\mathrm{sys}}$ . Thus, $\left[{Ĥ}_{\mathrm{sys}},L_{\omega }\right]=-\omega L_{\omega }$ and $\left[{Ĥ}_{\mathrm{sys}},L_{\omega }^{†}\right]=\omega L_{\omega }^{†}$ , showing that $L_{\omega }$ and act as effectively ladder operators for ${Ĥ}_{\mathrm{sys}}$ . The coupling constants are obtained from the real part of the Y−Y autocorrelation function and thus obey detailed balance: $\gamma_{\omega }=e^{\hbar \omega /k_{B}T}\gamma_{-\omega }$ . This relation forces the density matrix *ρ̂*(*t*) of the MME to decay towards the thermal equilibrium density matrix solution. We take a model function

30
$$\gamma_{\omega }=\gamma_{*}e^{1/2\hbar \omega /k_{B}T}$$

## The QMC Single Stochastic Propagation Step

Given a single-configuration ket

31
$$\left|n_{0}\right\rangle =\left|n_{1↑}\cdot \cdot \cdot n_{L↑},n_{1↓}\cdot \cdot \cdot n_{L↓}\right\rangle$$

32
$$\left|n_{0}\right\rangle =\left|n_{1↑}\cdot \cdot \cdot n_{L↑},n_{1↓}\cdot \cdot \cdot n_{L↓}\right\rangle$$

we construct a random ket equal to a product of another single-configuration ket |**n**_1_⟩ and an amplitude *a*_**n**_0_,**n**_1__, such that

33
$$e^{-Ĥ\Delta \beta }\left|n_{0}\right\rangle =E\left[\left|n_{1}\right\rangle a_{n_{0},n_{1}}\right]$$

### Basic Approach

The construction is based on the Suzuki-Trotter factorization and a first-order Taylor expansion

$$e^{-Ĥ\Delta \beta }=e^{-Û\Delta \beta /2}\left(1-\mathrm{T̂}\Delta \beta \right)e^{-Û\Delta \beta /2}+O\left(\Delta \beta^{2}\right)$$

Applying this to |**n**_0_⟩ yields

$$e^{-Ĥ\Delta \beta }\left|n_{0}\right\rangle \approx e^{-Û\Delta \beta /2}\left(1-\mathrm{T̂}\Delta \beta \right)\left|n_{0}\right\rangle e^{-U_{n}\Delta \beta /2}$$

where

$$e^{-Û\Delta \beta /2}\left|n_{0}\right\rangle =\left|n_{0}\right\rangle e^{-U_{n_{0}}\Delta \beta /2},U_{n_{0}}=U\sum_{i=1}^{L}n_{i↑}n_{i↓}$$

since |**n**_0_⟩ is an eigenket of Û .

Next, we rewrite

34
$$e^{-Û\Delta \beta /2}\left(1-\mathrm{T̂}\Delta \beta \right)\left|n_{0}\right\rangle =\left|n_{0}\right\rangle -\sum_{\left\langle \mathrm{ij}\right\rangle }C_{\left\langle \mathrm{ij}\right\rangle }{â}_{j\sigma }^{†}{â}_{i\sigma }\left|n_{0}\right\rangle$$

with non-negative coefficients

$$C_{\left\langle \mathrm{ij}\right\rangle }≔t\Delta \beta e^{\left(n_{i\mathrm{\sigma ̅}}-n_{j\mathrm{\sigma ̅}}\right)U\Delta \beta /2}n_{i\sigma }\left(1-n_{j\sigma }\right)$$

The factor *n*_*i*σ_(1–*n*_*j*σ_) equals 1 if site *i*σ is occupied and site *j*σ empty in |**n**_0_⟩ and 0 otherwise. Here σ̅ denotes the opposite spin of σ.

### Stochastic Representation

We now represent the operator action as an expectation value

35
$$e^{-Û\Delta \beta /2}\left(1-\mathrm{T̂}\Delta \beta \right)\left|n_{0}\right\rangle =E\left|\phi \right\rangle$$

where |ϕ⟩ is a *random* single configuration ket multiplied by a random amplitude. Let

$$l=\left(1+\sum_{\left\langle \mathrm{ij}\right\rangle }C_{\left\langle \mathrm{ij}\right\rangle }\right)$$

The sampling rules are

1. With probability $\frac{1}{l}$
  $$\left|\phi \right\rangle =\left|n_{0}\right\rangle l$$
2. With probability $\frac{C_{\left\langle \mathrm{ij}\right\rangle }}{l}$
  $$\left|\phi \right\rangle ={â}_{j\sigma }^{†}{â}_{i\sigma }\left|n_{0}\right\rangle \left(-l\right)$$

These probabilities minimize the variance of the stochastic estimator.

### Final Expression

Including the remaining diagonal factor, the random ket satisfying eq 33 is

36
$$\left|n_{1}\right\rangle a_{n_{0},n_{1}}≔\left|\phi \right\rangle e^{-U_{n_{0}}\Delta \beta /2}$$

## Notes

DFT and sDFT runs of H_8_ (TXT)
DFT and sDFT runs of H_128_ (TXT)
Coordinates of the atoms, the cutoff energies (TXT)
The (Baldereschi) k-point (TXT)
